# Complete Mitochondrial Genome of the Endemic Loach *Leptobotia tchangi* and Its Phylogenetic Placement Within Botiidae

**DOI:** 10.3390/genes17091087

**Published:** 2026-09-09

**Authors:** Yuting Hu, Guoqing Duan, Huaxing Zhou, Amei Liu, Huan Wang

**Affiliations:** 1 Fishery Institute of Anhui Academy of Agricultural Sciences, Hefei 230031, China; duanguoqing@aaas.org.cn (G.D.); zhouhuaxing@aaas.org.cn (H.Z.); liuamei@aaas.org.cn (A.L.); wanghuan@aaas.org.cn (H.W.); 2 Anhui Province Key Laboratory of Aquaculture & Stock Enhancement, Hefei 230031, China; 3 Agricultural Germplasm Resources Bank of Anhui Province, Hefei 230031, China

**Keywords:** *Leptobotia tchangi*, mitogenome, phylogeny, Botiidae, Leptobotiinae

## Abstract

Background: *Leptobotia tchangi* is a loach species endemic to South China, but its mitochondrial genome has not yet been characterized, limiting understanding of its evolutionary relationships and conservation genetics. Methods: Here, we report the first complete mitochondrial genome of *L. tchangi*, using next-generation sequencing, assembly and bioinformatics analyses. Results: The double-stranded circular mitogenome has 16,590 bp and contains: 13 protein-coding genes (PCGs), 2 ribosomal RNA genes, 22 transfer RNA genes, and a non-coding control region (D-loop) containing conserved ETAS and CSB motifs. The overall base composition is 25.0% thymine (T), 27.9% cytosine (C), 31.1% adenine (A), and 16.0% guanine (G), showing a clear A + T bias (56.1%) which is consistent with other Botiidae mitogenomes. To infer the phylogenetic placement of *L. tchangi* within Botiidae, we conducted both Bayesian inference and maximum-likelihood phylogenetic analyses based on the concatenated PCG sequences. Our results strongly support (1) the monophyly of the subfamilies Leptobotiinae and Botiinae, as well as the monophyly of each genus within the family Botiidae; (2) three sister-group relationships within Botiinae: (*Botia* + *Chromobotia*), (*Ambastaia* + *Sinibotia*), and (*Syncrossus* + *Yasuhikotakia*), with the latter two species groups also being sister groups; (3) *L. tchangi* being most closely related to *Leptobotia taeniops*. Conclusions: These findings not only provide essential molecular markers for the species identification and conservation genetics of *L. tchangi*, but also clarify the taxonomic status of *L. tchangi* within Botiidae.

## 1. Introduction

The family Botiidae (Cypriniformes), commonly known as loaches, represents a diverse group of benthic freshwater fishes predominantly distributed across East and Southeast Asia [1,2]. Currently comprising 62 recognized species (FishBase: https://www.fishbase.org, accessed on 31 July 2026), botiid loaches exhibit remarkable morphological diversity and occupy a wide range of freshwater habitats, from slow-moving lowland rivers to fast-flowing hillstreams [3,4]. In recent years, molecular phylogenetic studies have substantially revised the taxonomy of this family, establishing the subfamilies Leptobotiinae and Botiinae and clarifying the generic boundaries among previously conflated lineages [1,5]. Despite these advances, however, complete mitogenomic resources remain lacking for many endemic species, particularly within the genus *Leptobotia*, hindering comprehensive assessments of their evolutionary relationships and conservation genetic status.

*Leptobotia* is a genus within the subfamily Leptobotiinae, comprising small to medium-sized benthic freshwater fishes endemic to China that inhabit fast-flowing hillstreams and major river systems [1,3]. It includes 19 valid species (FishBase: https://www.fishbase.org, accessed on 30 July 2026), primarily distributed in the Yangtze and Pearl River basins, as well as coastal rivers in Zhejiang Province and Fujian Province [1,2,4,6,7]. Two species, *Leptobotia flavolineata* Wang, 1981 and *Leptobotia orientalis* Xu, Fang & Wang, 1981, extend northward to the Haihe River Basin in China [8,9]. Despite its considerable species richness, the taxonomy of the genus remains challenging due to morphological conservatism and limited molecular data, particularly at the mitogenomic level.

*L. tchangi* Fang, 1936 is a species endemic to South China. Its type locality was designated as the Qiantang River in Tianmu Mountain, Zhejiang Province [10], and it has also been recorded in the Yuanjiang River (Hunan Province), a tributary of the middle Yangtze River [11], as well as in the Huangshan region of Anhui Province (the upper reaches of the Qiantang River system) and Xinjiang River (Jiangxi Province), also a tributary of the middle Yangtze River [12,13]. This species prefers shallow riffle habitats with relatively fast current and gravel or pebble substrate [14,15]. Given its restricted distribution and the vulnerability of its preferred microhabitats to anthropogenic disturbances [15], *L. tchangi* may be vulnerable to population declines, although direct empirical evidence remains scarce. However, the lack of genetic resources has hindered the formulation of effective conservation strategies, particularly in delineating its evolutionary significant units (ESUs) and assessing population genetic diversity.

Phylogenetic relationships of *L. tchangi* inferred from short mitochondrial fragments have been notably unstable. Early studies by Tang et al. [12,13,16,17], using the D-loop or cytochrome *b* (*cytb*) or D-loop + *cytb* sequences separately within Botiinae, consistently recovered *L. tchangi* as sister to *Leptobotia taeniops* (Sauvage, 1878) by Bayesian inference (BI), maximum parsimony (MP) or maximum likelihood (ML) methods, with *Leptobotia rubrilabris* (Dabry de Thiersant, 1872) as the next closest relative, although most nodal support values remained modest. More recently, Guo and Zhang (2021) [6] and Guo et al. (2023) [7] incorporated additional species, including *Leptobotia hengyangensis* (Huang & Zhang, 1986), into their phylogenetic analyses. Based on analysis of the *cytb* dataset by the BI method, Guo and Zhang (2021) [6] recovered *L. tchangi* as sister to *L. hengyangensis*, with the *L. taeniops* + *L. rubrilabris* clade as their sister group. In contrast, Guo et al. (2023) [7], analyzing combined cytochrome c oxidase subunit I (*cox1*) and *cytb* data by the BI method, found that *L. tchangi* first clustered with *L. hengyangensis* as sister species, then together formed a clade with *L. taeniops*, and subsequently with *L. rubrilabris*. Such profound discordance among single-locus or combined short-marker datasets highlights a critical limitation: these markers are prone to stochastic errors, base-compositional saturation at deeper nodes, and a paucity of phylogenetically informative characters, particularly for resolving recent divergences [18,19].

In contrast, the nearly complete set of genetic information from a whole mitogenome provides a substantially larger and more heterogeneous dataset, offering greater statistical support and robustness for phylogenetic reconstruction at both shallow and deep taxonomic levels. Complete mitogenomes have been successfully employed to resolve contentious relationships within various fish groups, including Cypriniformes [18,19,20,21], and are increasingly recognized as a valuable resource for mitochondrial phylogenetics and comparative genomics [18,22]. Moreover, comparative mitogenomic analyses—including gene order, structural features, and codon usage patterns—can provide additional evolutionary insights beyond traditional sequence-based approaches [18,19,20,21,22]. To date, however, the complete mitogenome of *L. tchangi* has remained uncharacterized, representing a significant gap in our understanding of its evolutionary biology and hindering robust phylogenetic placement within Botiidae.

In this study, we sequenced and assembled the complete mitochondrial genome of *L. tchangi* using next-generation sequencing (NGS) technology. Through comparative genomic analyses with closely related species, we aim to (i) provide robust molecular evidence for the taxonomic status of *L. tchangi*, (ii) resolve the conflicting phylogenetic hypotheses within Botiidae, and (iii) evaluate the utility of mitogenomic data in clarifying recent divergences in this group. Our findings are expected to contribute to a phylogenetically informed framework for the conservation of this endemic and potentially vulnerable species.

## 2. Materials and Methods

### 2.1. Sample Collection and Preservation

In August 2025, three specimens of *L. tchangi* were collected using cage traps from the shallow, fast-flowing reaches of the Qiantang River in Huangshan City, Anhui Province, China (29.91° N, 118.11° E). This sampling site was selected because it lies within the same drainage basin as the type locality and represents a known extant population of this species as documented in previous surveys [12,13]. All specimens were morphologically identified following the diagnostic characters in the original description [10], e.g., body coloration pattern, scales on the cheek, and the absence of a pair of fleshy protuberances on the chin. The fish were euthanized with an overdose of clove oil solution (100 mg/L), following ethical guidelines for aquatic animal research and in accordance with the ethical regulations of the Anhui Academy of Agricultural Sciences (Institutional Review Board approval No. AAAS2025-21, approved on 20 August 2025). Subsequently, two specimens were fixed in 10% neutral-buffered formalin for subsequent morphological verification, while the third specimen was immediately preserved in 95% ethanol for molecular analyses. All specimens are deposited in the Fish Specimen Collection at the Fishery Institute of the Anhui Academy of Agricultural Sciences.

### 2.2. DNA Extraction, Library Preparation and NGS

Genomic DNA was extracted from ~0.20 g muscle tissue from an adult *L. tchangi* using the MagPure Bacterial DNA Kit (Magen, Guangzhou, China) following the manufacturer’s protocol. DNA quality and concentration were assessed using a Qubit 4.0 fluorometer (Thermo Fisher Scientific, Waltham, MA, USA) with the dsDNA HS Assay Kit (Sangon, Shanghai, China) and verified by 1% agarose gel electrophoresis (AGE). A total of 500 ng of high-quality genomic DNA was fragmented using a Covaris M220 (Covaris, Woburn, MA, USA) to generate inserts of ~400 bp. The fragmented DNA was then used for library construction with the Hieff NGS^®^ MaxUp II DNA Library Prep Kit (YEASEN, Shanghai, China). The library preparation followed the manufacturer’s recommended protocol, which included end-repair, A-tailing, and adaptor ligation. Adapter-ligated fragments were then amplified by PCR. The PCR reaction mixture (50 µL total volume) consisted of 25 µL of 2× Super Canace^®^ II High-Fidelity Master Mix, 5 µL of Primer Mix (provided in the kit), and the entire volume of the adapter-ligated DNA sample. Thermal cycling was performed under the following conditions: initial denaturation at 98 °C for 30 s; followed by 12 cycles of denaturation at 98 °C for 10 s, and annealing/extension at 65 °C for 75 s; with a final extension at 65 °C for 5 min. The amplified library was purified and size-selected (~400 bp) using Agencourt AMPure XP beads (Beckman Coulter, Brea, CA, USA). Library quality and concentration were confirmed using a Qubit 4.0 fluorometer and 2% AGE, respectively. The pooled library was sequenced on the DNBSEQ-T7 platform (BGI, Shenzhen, China) with 150 bp paired-end reads.

### 2.3. Sequence Assembly, Annotation and Validation

Raw reads were trimmed using Fastp v0.11.2 [23] and assembled into contigs with SPAdes v3.15 [24] with default parameters. The complete mitogenome was then assembled using MITObim v1.9.1 [25] with the *L. taeniops* mitogenome (NC026130) as the seed sequence and 30 iterations. The assembled mitogenome was annotated using the MITOS2 web server (http://mitos2.bioinf.uni-leipzig.de/, accessed on 14 May 2026) with the ‘Vertebrate Mitochondrial’ genetic code and the RefSeq 89 Metazoa reference database [26]. Annotation was manually curated in Geneious Prime 2025.1 (https://www.geneious.com, accessed on 15 May 2026) to verify start/stop codons. The circular mitogenome map was visualized using MitoDraw [27]. To validate the assembly, the *cytb* gene and the D-loop region were independently amplified and Sanger-sequenced using universal primers for Cypriniformes (Supplementary Table S1) [28,29]. PCR products were sequenced bidirectionally, and the resulting sequences were compared with the assembled mitogenome in MEGA 12.09 [30], confirming 100% sequence identity.

### 2.4. Bioinformatics Analysis

Nucleotide composition, AT and GC skew values, codon preference indices, and relative synonymous codon usage (RSCU) in each protein-coding gene (PCG) were calculated using PhyloSuite v2 [31] with default parameters. The AT-skew and GC-skew were computed following the formulas: AT-skew = (A − T)/(A + T) and GC-skew = (G − C)/(G + C). The structural characteristics of the D-loop region, particularly the conserved ETAS and CSB motifs, were identified through multiple sequence alignment against previously characterized Botiidae mitogenomes [12] using MAFFT v7.505 [32] and the L-INS-i strategy, with manual verification of motif boundaries based on sequence similarity and positional conservation.

To evaluate selective pressure acting on each PCG, the mean nonsynonymous substitution (Ka), synonymous substitution rate (Ks), and their ratio (Ka/Ks) were estimated using DnaSP 6.0 [33]. Pairwise comparisons were conducted across all 32 Botiinae mitogenome sequences (including *L. tchangi*) (Table 1). Only alignments with no internal stop codons or frameshifts were retained for Ka/Ks estimation; alignments containing ambiguous sites were excluded from pairwise comparisons to avoid bias.

Genetic distances among the 32 Botiinae sequences were calculated using MEGA 12.09 [30] based on the Kimura 2-parameter (K2P) model, which was selected for its appropriateness for mitochondrial PCGs with moderate sequence divergence and its widespread use in comparative mitogenomic studies within Cypriniformes [12,34]. Distance matrices were computed for the concatenated 13 PCG dataset. The sequence alignments used for distance and selection analyses were generated following the alignment procedure described in Section 2.5, with gap positions treated as pairwise deletion in MEGA analyses.

### 2.5. Phylogenetic Analysis

We retrieved all available mitochondrial genome sequences for the family Botiidae from the GenBank database. To ensure taxonomic representativeness, only one sequence per species was retained, with priority given to reference sequences bearing the “NC” accession prefix. Following previous studies [34], sequences suspected of misclassification at the species level within the subfamily Leptobotiinae were excluded. After filtering, a final dataset of 35 mitogenome sequences was obtained, comprising 32 Botiidae species (including *L. tchangi*) and three Cobitidae species as outgroups (Table 1). This dataset was aligned using MAFFT v7.505 [32] with default parameters, and the 13 PCGs were extracted and aligned separately. To avoid frameshift errors and ensure codon-aware gap placement, the PCG alignments were further refined using MACSE v2 [35].

Phylogenetic analysis were performed on the concatenated 13 PCGs (11,391–11,409 bp). The optimal partitioning schemes and best-fit substitution models were selected using ModelFinder v2.2.0 [36] under the Bayesian Information Criterion (BIC) for maximum-likelihood (ML) analysis, and PartitionFinder v2 [37] under the Akaike Information Criterion corrected (AICc) for Bayesian inference (BI), both employing a gene + codon partitioning strategy with edge-linked branch lengths. ML trees were constructed using IQ-TREE v2.4.0 [38] with 1000 bootstrap replicates, and BI trees were generated with MrBayes v3.2.6 [39] running two independent runs of four Markov chains for 5 million generations, sampling every 1000 generations, with a burn-in fraction of 25%. All trees were visualized using iTOL (https://itol.embl.de/, accessed 30 July 2026).

## 3. Results

### 3.1. Mitochondrial Genomic Organization and Composition of L. tchangi

**Figure 1:**
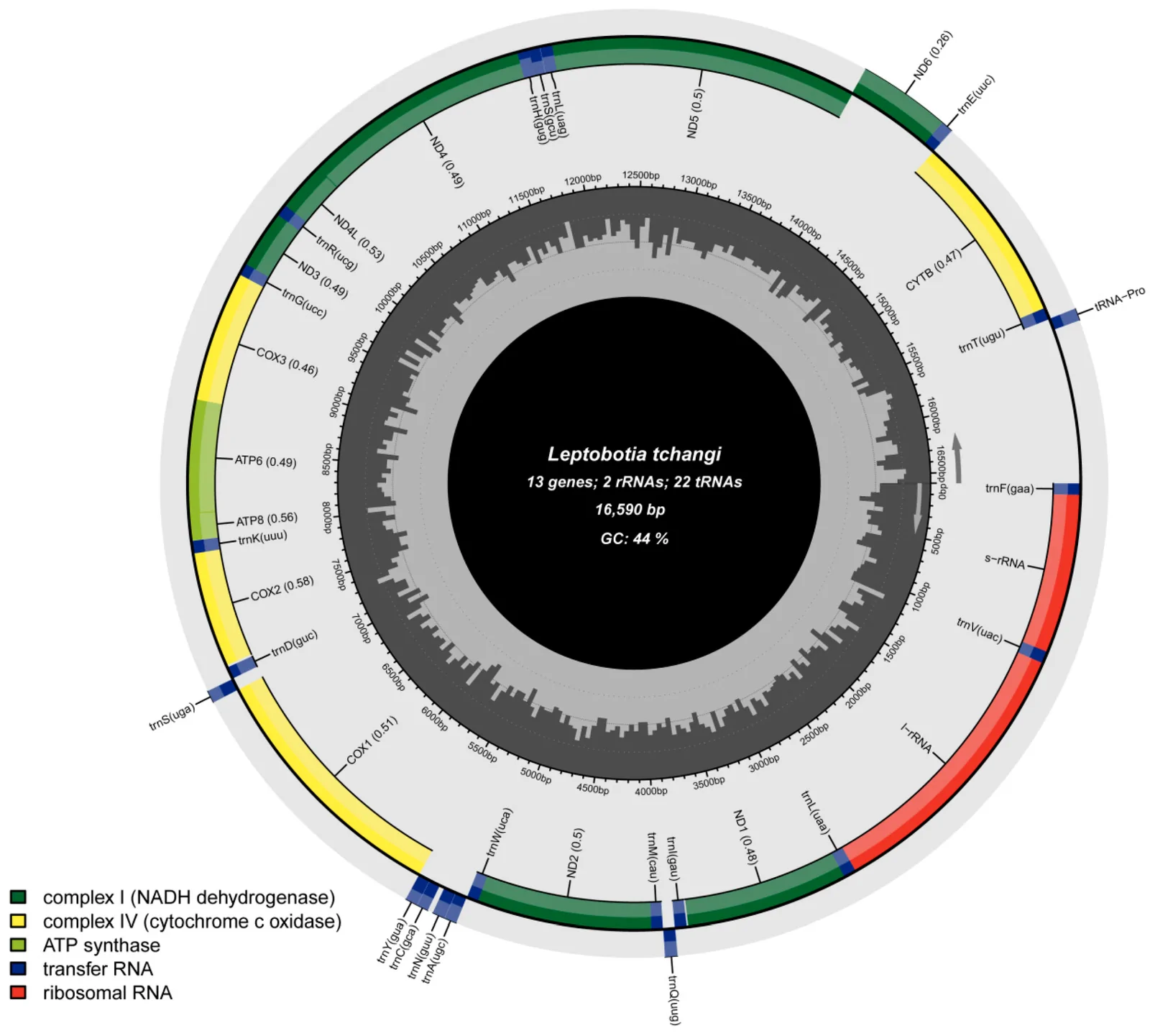
Circular map of the mitochondrial genome of *L. tchangi*. From inside to outside: GC content, base sequencing depth, and genes. In the outermost genes, inner circle contains genes transcribed in the forward direction, while the outer circle contains genes transcribed in the reverse direction.

A total of 11.58 Gb of raw reads and 11.39 Gb of clean reads were generated (Supplementary Table S2), yielding a high-quality circular mitogenome of 16,590 bp (GenBank: PZ213610), with an average coverage of ~1256× (Supplementary Table S3). It contains the typical set of 37 vertebrate mitochondrial genes: 13 PCGs, two ribosomal RNA genes (12S rRNA and 16S rRNA), 22 transfer RNA genes (tRNAs), and a non-coding control region (D-loop) (Figure 1, Table 2). Most genes are encoded on the heavy strand, whereas *nad6* and 8 tRNAs are located on the light strand. For the 13 PCGs, ATG is the start codon except for *cox1* (GTG). Seven PCGs terminate with a complete TAA stop codon, while others use incomplete stop codons (TA for 3 genes and a single T for 3 genes). The 22 tRNAs is 66 to 76 bp in length, with tRNA-Cys being the shortest and tRNA-Leu (UUR) the longest. The 12S rRNA (954 bp) and 16S rRNA (1681 bp) are situated between typical tRNA gene clusters.

The D-loop (926 bp in size) is flanked by tRNA-Pro and tRNA-Phe (Figure 1). It contains 7 conserved regulatory motifs: one extended terminal associated sequence (ETAS), a central conserved domain comprising CSB-F, CSB-E, and CSB-D, and a conserved sequence block (CSB) region composed of CSB-1, CSB-2, and CSB-3 (Supplementary Figure S1). Thirteen intergenic spacers (1–30 bp) were detected in the mitogenome, with the largest one (30 bp) positioned between tRNA-Asn and tRNA-Cys. Within this spacer, a putative origin for light-strand replication (OL) was predicted, forming a stem-loop structure with a conserved 10-bp loop motif (Supplementary Figure S2). Additionally, 6 gene overlaps (1–10 bp) were found, with the largest overlap (10 bp) occurring between *atp8*/*atp6* and *nad4L*/*nad4*.

The overall base composition is 25.0%T, 27.9%C, 31.1%A, and 16.0%G, with a pronounced A + T bias of 56.1% (Table 3). The D-loop (926 bp) exhibits the highest A + T content (68.2%), consistent with its low selective constraint. Among the three codon positions of the 13 PCGs, the A + T content increases progressively from the first (47.8%) to the third position (61.2%). Strand-specific skew analysis revealed a negative GC-skew across most genomic regions, with the exception of *nad6* and tRNAs, which show a positive GC-skew (Table 3). However, the AT-skew is negative for *cox1*, *nad3*, *nad4L*, and *nad6*, yet positive for the full mitogenome, rRNAs, tRNAs, the D-loop, concatenated PCGs, and the other nine genes.

### 3.2. Mitochondrial Codon Usage Patterns of L. tchangi

**Figure 2:**
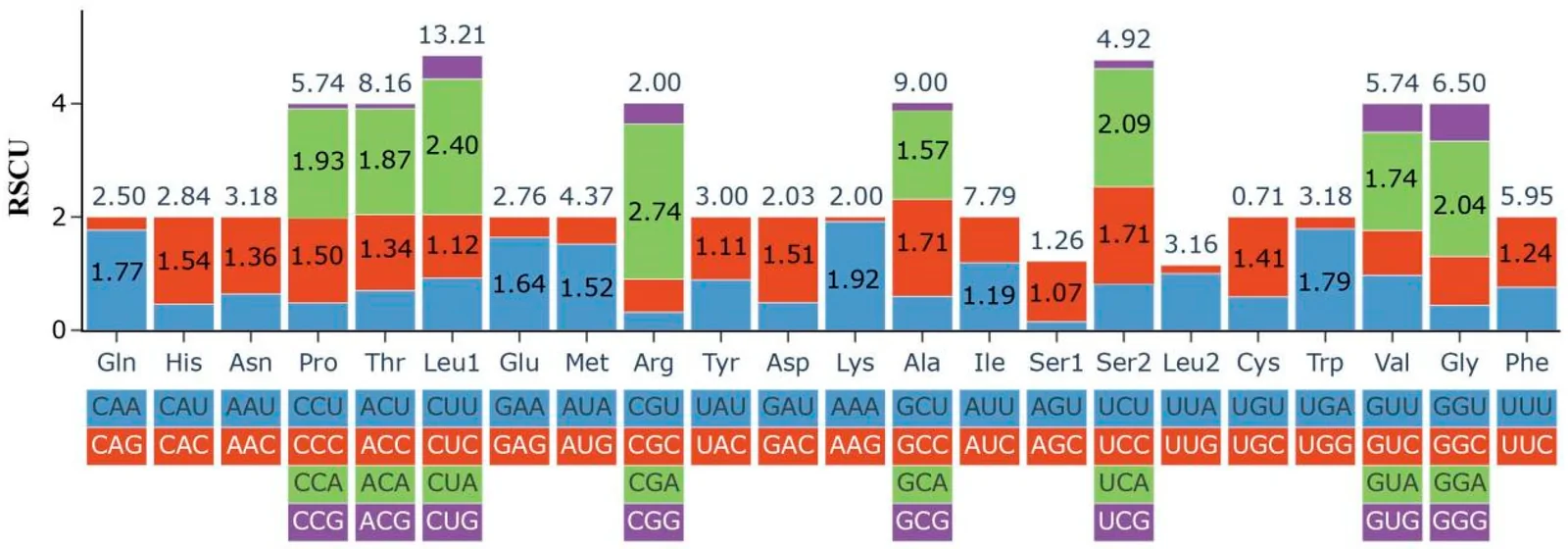
Amino acid percentages (upper,%) and RSCU values > 1 (inside) in the 13 PCGs of the *L. tchangi* mitogenome.

Analysis of the 13 PCGs revealed a strong AT-rich codon usage bias. Excluding stop codons, the PCGs encode 3800 amino acids, of which Leucine (16.37%) is the most abundant and Cysteine (0.71%) the least frequent (Figure 2). Relative synonymous codon usage (RSCU) analysis identified 26 preferred codons (RSCU > 1.0), with a marked preference for A/T-ending synonymous codons (Figure 2, Table 4). Notably, four codons—CGA (2.74), CUA (2.40), UCA (2.09), and GGA (2.04)—exhibited RSCU values exceeding 2.0, reinforcing the strong bias toward adenine at the third codon position. In addition, in the vertebrate mitochondrial genetic code, UGA is recognized as Tryptophan (Trp) and was used far more frequently (RSCU = 1.79) than its synonym UGG (RSCU = 0.21) (Figure 2, Table 4).

### 3.3. Nucleotide Diversity of Mitochondrial PCGs in Botiidae

**Figure 3:**
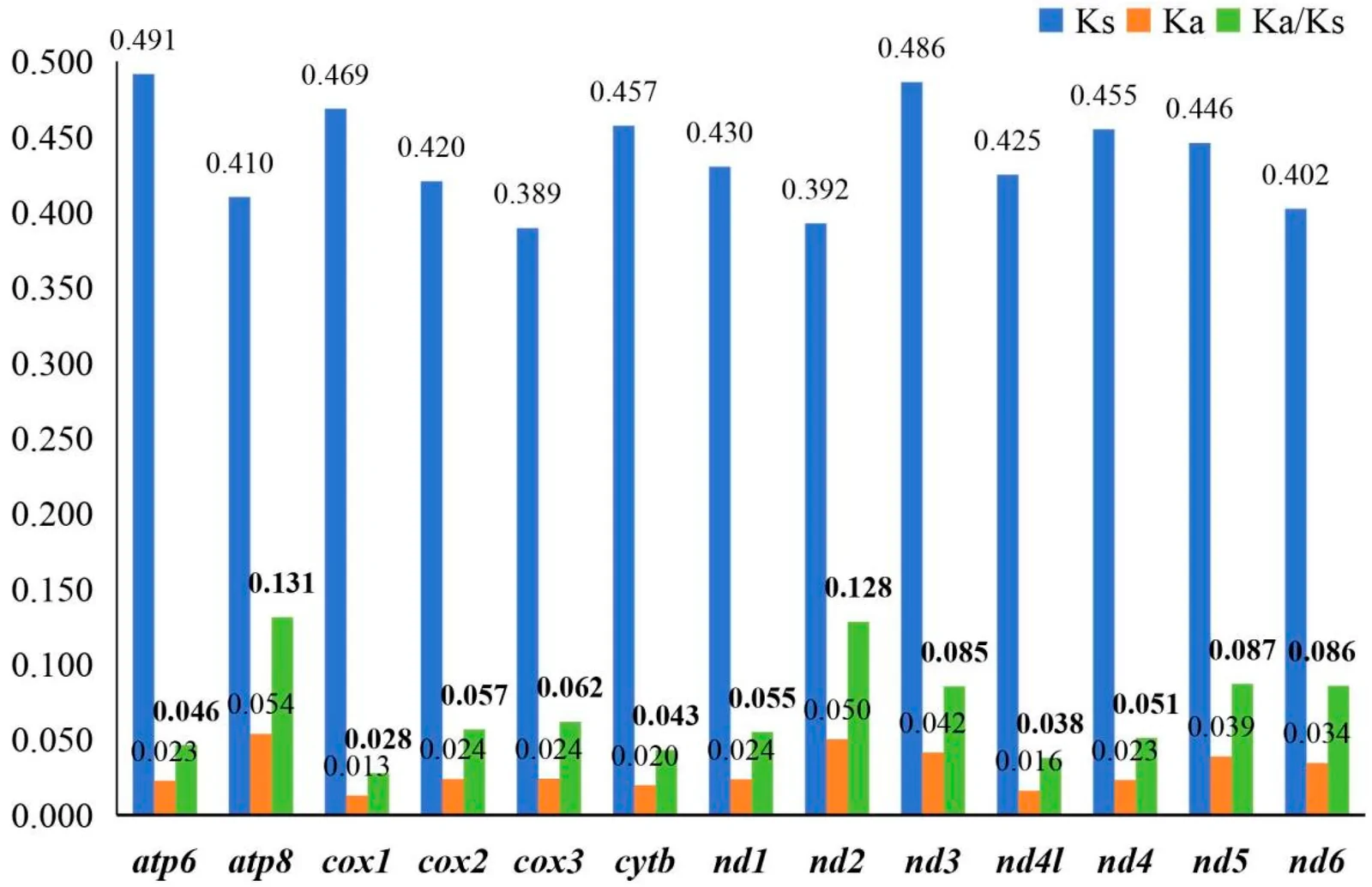
The mean Ka, Ks, and Ka/Ks values of 13 PCGs of 32 Botiidae species.

The synonymous substitution rates (Ks) of the 13 mitochondrial PCGs in the family Botiidae averaged 0.435, ranging from 0.389 (*cox3*) to 0.491 (*atp6*), while the nonsynonymous substitution rates (Ka) averaged 0.030, ranging from 0.013 (*cox1*) to 0.054 (*atp8*) (Figure 3). The Ka/Ks ratios for all 13 PCGs were substantially less than 1, ranging from 0.028 (*cox1*) to 0.131 (*atp8*) (Figure 3). Among these, atp8 (Ka/Ks = 0.131) and nd2 (Ka/Ks = 0.128) exhibited the highest ratios compared to the other 11 genes (≤0.087).

### 3.4. Phylogenetic Relationship

**Figure 4:**
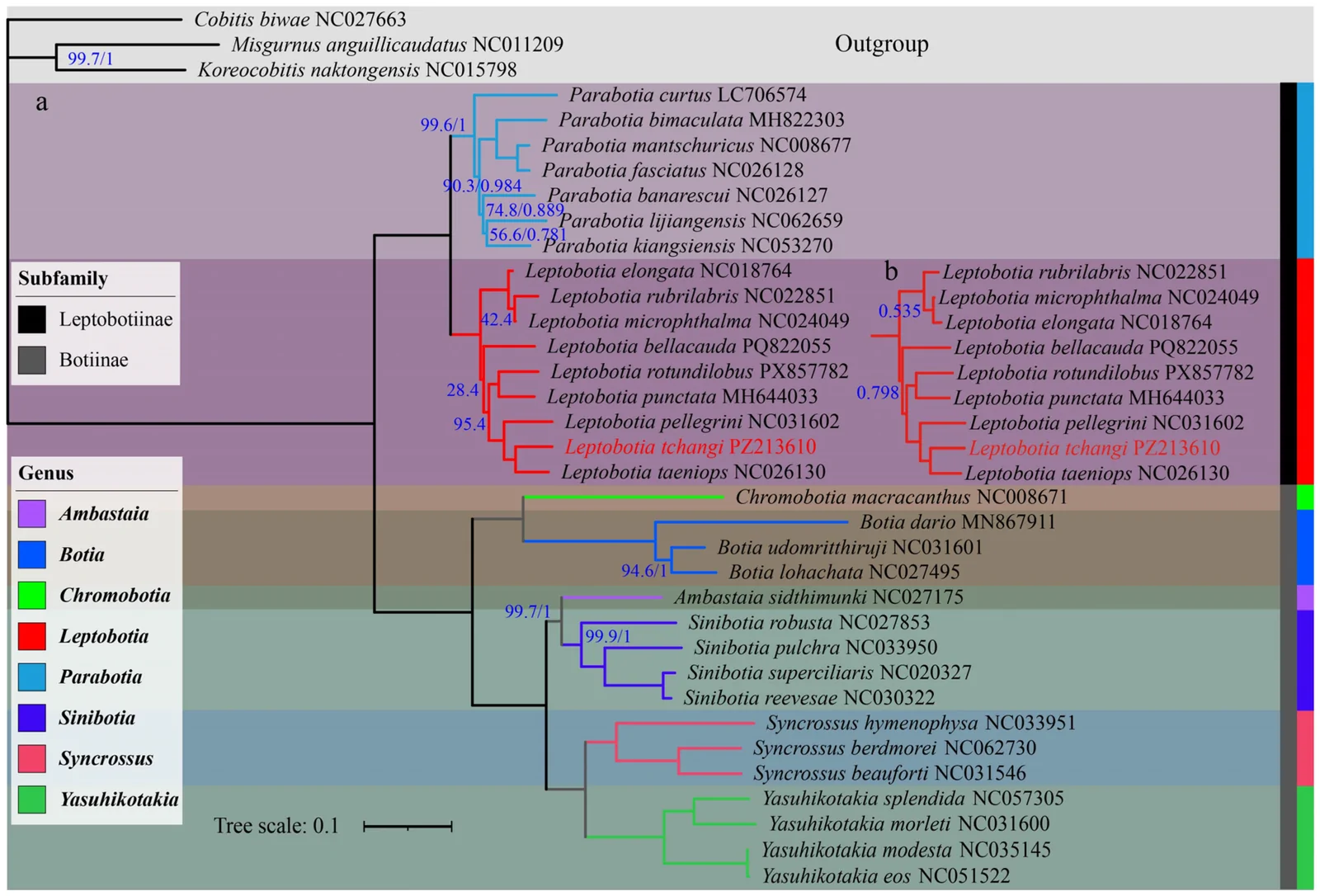
Phylogenetic tree reconstructed using maximum-likelihood (ML) (**a**) and Bayesian inference (BI) methods (**b**) based on 13 PCGs of 32 Botiidae species and 3 outgroups. Values at nodes represent bootstrap values (left) and posterior probabilities (right), with unlabelled nodes indicating maximal support (100%/1.00). The target species of this study are highlighted in red.

Phylogenetic trees reconstructed using ML and BI analyses with the best partitioning schemes and substitution models (Supplementary Table S4) yielded largely congruent topologies, with full support for the monophyly of the family Botiidae, its two subfamilies (Leptobotiinae and Botiinae), and all constituent genera (Figure 4). Within the subfamily Leptobotiinae, our analyses robustly resolved *L. tchangi* as the sister species to *L. taeniops* with maximum support, a relationship that has been contentious in previous studies using short mitochondrial markers. Within Botiinae, the phylogenetic framework was also well-resolved, identifying three robust sister-group relationships: (*Botia* + *Chromobotia*), (*Ambastaia* + *Sinibotia*), and (*Syncrossus* + *Yasuhikotakia*), all with support values exceeding 99%. The only discordant node between the ML and BI trees involved the placement of *Leptobotia elongata* (Bleeker, 1870) and *L. rubrilabris*, which were reciprocally exchanged with insignificant support, suggesting a rapid radiation or limited phylogenetic signal within this subclade.

Pairwise genetic distance analysis within genus based on the 13 PCGs revealed an exceptionally low K2P distance (0.16%) between *Y. modesta* (Bleeker, 1864) and *Y. eos* (Taki, 1972), which is substantially lower than the interspecific distances observed among the genus *Yasuhikotakia* (9.09–12.08%, exclude this paired species) or other congeners within Botiidae (1.23–15.27%) (Supplementary Table S5) and falls within the range typically expected for intraspecific variation in fishes [40]. Additionally, low interspecific genetic distances (≤3.65%) were also found among several species pairs (Table S5), including *Sinibotia reevesae* (Chang, 1944) vs. *Sinibotia superciliaris* Günther, 1892 (2.01%), *Parabotia fasciatus* Dabry de Thiersant, 1872 vs. *Parabotia mantschuricus* (Berg, 1907) (2.37%), and among *Leptobotia microphthalma* Fu & Ye, 1983, *L. elongata*, and *L. rubrilabris* (1.23–3.65%), which are all lower than distances typically observed between other species within their respective genera.

## 4. Discussion

### 4.1. General Features of the L. tchangi Mitogenome

The complete mitogenome of *L. tchangi* (16,590 bp) exhibits the canonical vertebrate gene content and organization, with its genome size being consistent with those of other *Leptobotia* species, all approximately 16,590 bp (Table 1). The overall A + T bias (56.1%), with the highest value in the D-loop (68.2%), and the strand-specific skew patterns are consistent with those reported for other Botiidae mitogenomes, especially for other *Leptobotia* species (Table 1) [34,41,42,43,44].

Notable structural features that were identified include a putative light-strand replication origin (OL) within the largest intergenic spacer (tRNA-Asn and tRNA-Cys) and conserved 10-bp overlaps between *atp8*/*atp6* and *nad4L*/*nad4*, both of which are also present in *L. rotundilobus* [34] and *L. elongata* [44], with the overlaps likely facilitating translational coupling of these functionally related genes. The D-loop contains all three conserved regulatory domains (ETAS, CD with CSB-F/E/D, and CSB-1/2/3), which is widely documented in diverse members of Botiidae [12], with notable instances observed in *L. rotundilobus* [34], indicating that, despite the high variability of this region, its replication and transcriptional regulatory functions are conserved within Botiidae.

### 4.2. Codon Usage Bias and Purifying Selection

Analysis of codon usage in the 13 PCGs of *L. tchangi* revealed a strong preference for A/T-ending synonymous codons (e.g., CUA, UCA, CGA, and GGA). This pattern is consistent with the mutational pressure toward A/T enrichment, a hallmark of vertebrate mitochondrial genomes, and the RSCU values suggest that purifying selection is the dominant force shaping synonymous codon usage [45]. The preferential use of UGA over UGG for encoding Tryptophan further reflects the streamlined translational machinery of the vertebrate mitochondrial genetic code [46].

To investigate the evolutionary constraints on mitochondrial genes at the family level, we calculated the Ka/Ks ratios across Botiidae. All 13 PCGs exhibited Ka/Ks ratios substantially lower than 1 (0.028–0.131), indicating that purifying selection is the predominant evolutionary force acting on the entire mitochondrial protein-coding genome. Notably, *atp8* (Ka/Ks = 0.131) and *nad2* (0.128) showed the highest ratios, suggesting relatively relaxed functional constraints. While the elevated ratio for *atp8* is expected given its small size and accessory role in ATP synthase, the higher ratio for *nad2* warrants particular attention.

Given the broad ecological distribution of Botiidae, which inhabits diverse freshwater environments ranging from slow-flowing lowland rivers to fast-flowing hillstreams [3,4]. we propose that this elevated ratio in *nad2* warrants particular attention. We tentatively suggest that it may reflect functional adaptation to benthic, flowing-water habitats with variable oxygen availability [14,15], rather than merely relaxed constraint. Similar patterns of elevated *nad2* evolutionary rates have been documented in other fishes: positive selection on *nad2* has been detected in the high-altitude plateau loach *Triplophysa brevicauda* (Herzenstein, 1888), where it is interpreted as a molecular adaptation linked to high energy demands and regional endothermy [47], as well as in billfishes [48]. Nevertheless, alternative explanations—such as differences in effective population size or mutation rate heterogeneity among genes—cannot be ruled out, and rigorous testing of this hypothesis would require environmental association analyses and functional experiments.

### 4.3. Phylogenetic Implications

The phylogenetic trees (ML and BI tree) are largely congruent with the topologies obtained in previous molecular studies on Botiidae [16,17,49], further confirming the monophyly of Leptobotiinae and Botiinae, as well as that of each genus within the family. The only discordance between our two trees—the reciprocal placement of *L. elongata* and *L. rubrilabris*—is minor and does not affect the overall backbone, but it does hint at potential instability within *Leptobotia*, a genus that has historically posed taxonomic challenges [6,7,17,50].

Most importantly, our analyses robustly resolved *L. tchangi* as the sister species to *L. taeniops* with maximum support (100%), thereby clarifying the long-controversial phylogenetic position of this endemic species. This result aligns with the finding of Tang et al. [12,13,16,17] based on D-loop and *cytb* data, which also recovered this sister relationship. Notably, our dataset comprising 13 concatenated PCGs (approximately 11,400 bp) provides substantially more informative sites than the single-locus or short-marker datasets (<1500 bp) used in previous conflicting studies [6,7], which are prone to stochastic errors, base-compositional saturation, and a paucity of phylogenetically informative characters [18,19]. This underscores that the previously observed topological instability was largely an artifact of limited molecular data rather than genuine biological discordance.

It should be noted that *L. hengyangensis* was not included in our phylogenetic analyses, because the only sequence currently available in GenBank (NC061212) was identified as a misidentification at the species level following previous studies [34] and was therefore excluded from our dataset to avoid potential biases. This precaution ensures that our phylogenetic inference reflects genuine species-level relationships rather than artifacts due to erroneous taxonomic assignment.

### 4.4. Taxonomic Implications of the Low Genetic Distance

The exceptionally low K2P distance between *Y. modesta* and *Y. eos* (0.16%) is far below the interspecific divergence range among the genus *Yasuhikotakia* (9.09–12.08%, excluding this paired species) or other congeners within Botiidae (1.23–15.27%) (Table S5), and is even comparable to, or lower than, the intraspecific genetic distance reported for many fish species [34,40]. This anomaly therefore warrants careful scrutiny. While this finding could theoretically reflect genuine biological phenomena such as recent speciation with incomplete lineage sorting [51] or undetected synonymy, the most parsimonious explanation is species misidentification of at least one of the GenBank sequences. Species misidentification in public sequence databases is not uncommon, particularly for morphologically similar taxa or when voucher specimens are unavailable for direct verification [34,52]. Carné Constans et al. [53] found that 32% of re-sequenced vouchers in GenBank yielded unequal sequences, with divergences reaching up to 33.95%, highlighting substantial errors at the most fundamental level of data curation. Similar issues have been documented specifically in fishes, where large-scale screening identified over 1300 problematic *cytb* sequences attributable to taxonomic misidentification or other errors [54].

The morphological similarity between *Y. eos* and *Y. modesta* exacerbates this problem, as the two species are readily confused, especially in juvenile or incomplete specimens [55]. Given that mitochondrial markers alone are insufficient to resolve recent divergences and are vulnerable to undetected sequencing errors or sample mix-ups [56], we recommend that future studies re-examine the voucher specimens corresponding to these sequences and employ nuclear markers to validate species boundaries within *Yasuhikotakia*. In contrast to the *Yasuhikotakia* case (0.16%), the other three low-distance comparisons (1.23–3.65%) (Supplementary Table S5) are less extreme and may simply reflect genuine interspecific divergence.

### 4.5. Biogeographic Perspectives

The robust sister relationship between *L. tchangi* and *L. taeniops* invites biogeographic interpretation. While *L. taeniops* has a broad distribution across the upper, middle, and lower Yangtze River and the Huai River basin [34], *L. tchangi* exhibits a more fragmented pattern, occurring in the Qiantang River (an independent basin) [10], as well as in the Yuanjiang River (tributaries of the middle Yangtze) [11,12,13]. This trans-basin disjunction pattern becomes even more intriguing when considering the distribution of *L. hengyangensis*, a close relative not included in our molecular analysis. *L. hengyangensis* is recorded in the Xiangjiang and Ganjiang rivers [57,58]—which are hydrologically linked to the Yuanjiang and Xinjiang via Dongting and Poyang lakes, respectively—mirroring the distributional pattern of *L. tchangi*. This repeated spatial arrangement tentatively suggests that Quaternary paleo-hydrological fluctuations, possibly combined with historical river captures or Pleistocene sea-level regressions, may have facilitated dispersal and vicariance events within the *L. tchangi*–*L. hengyangensis*–*L. taeniops* complex.

Beyond this specific species complex, the distributional patterns observed in *Leptobotia* may reflect a more general biogeographic dynamic. Four other species—*L. elongata*, *L. rubrilabris*, *L. microphthalma*, and *L. taeniops*—are distributed in the upper Yangtze River basin, with the first three largely restricted to this region, whereas L. taeniops extends into more regions, as mentioned earlier [34]. The close genetic affinities among these taxa, combined with the complex mosaic of sympatric and allopatric distributions within the genus, raise the possibility that they represent recently diverged species or a species complex undergoing active speciation—a scenario already documented in another congener, *Leptobotia citrauratea* (Nichols, 1925) [50]. Collectively, these lines of evidence suggest that the biogeographic history of *Leptobotia* has been shaped by both paleo-hydrological disturbances and recent speciation dynamics. Rigorous testing of these hypotheses, however, will require denser taxon sampling (particularly verified sequences of *L. hengyangensis*), genome-wide markers, and divergence time estimation.

### 4.6. Limitations and Future Directions

Several limitations of this study should be acknowledged. First, although our mitogenomic dataset provided robust resolution of the *L. tchangi*–*L. taeniops* sister relationship, the exclusion of *L. hengyangensis* due to sequence uncertainty prevents a full assessment of the phylogenetic relationships among all *Leptobotia* species. The topological differences between our study and those of Guo et al. [6,7]—which placed *L. hengyangensis* as sister to *L. tchangi*—cannot be definitively resolved until reliable sequences of *L. hengyangensis* from verified voucher specimens become available. Second, mitochondrial genomes, while powerful for phylogenetic reconstruction, represent a single linked genetic locus and may not fully capture the complexity of species histories, particularly in the presence of incomplete lineage sorting or hybridization [59]. Third, our limited geographic sampling (one specimen from the Qiantang River) precludes population-level genetic assessments of *L. tchangi*, which is essential for evaluating its conservation status and identifying Evolutionarily Significant Units.

Future studies should prioritize (i) obtaining reliable sequences of *L. hengyangensis* from topotypic specimens to clarify its phylogenetic position; (ii) employing genome-wide markers (e.g., SNPs or RAD-seq) to provide independent evidence for species relationships and to detect potential introgression; (iii) conducting comprehensive geographic sampling across the middle Yangtze lake systems (Dongting and Poyang Lake) and adjacent independent basins (e.g., Qiantang River) to assess population genetic diversity and phylogeographic structure; and (iv) integrating ecological niche modeling and morphological analyses to elucidate the drivers of diversification and adaptive evolution within *Leptobotia*. Such an integrative framework will not only deepen our understanding of the evolutionary history of this endemic genus, but also provide a solid scientific basis for conservation strategies targeting these vulnerable hillstream fishes.

## Figures and Tables

**Table 1:** Species, GenBank accession, and base-composition features of the 35 mitogenomes.

NO.	Species	Author	Accession NO.	Size (bp)	AT%	AT Skew	GC Skew
1	*Leptobotia bellacauda*	Bohlen & Slechtová, 2016	PQ822055	16,591	55.0	0.113	−0.272
2	*Leptobotia elongata*	(Bleeker, 1870)	NC018764	16,591	55.6	0.108	−0.272
3	*Leptobotia microphthalma*	Fu & Ye, 1983	NC024049	16,512	55.6	0.109	−0.274
4	*Leptobotia pellegrini*	Fang, 1936	NC031602	16,595	55.5	0.117	−0.281
5	*Leptobotia punctata*	Bohlen & Šlechtová, 2017	MH644033	16,593	55.8	0.107	−0.271
6	*Leptobotia rotundilobus*	Guo, Cao & Zhang, 2023	PX857782	16,593	55.2	0.115	−0.276
7	*Leptobotia rubrilabris*	(Dabry de Thiersant, 1872)	NC022851	16,585	55.9	0.109	−0.272
8	*Leptobotia taeniops*	(Sauvage, 1878)	NC026130	16,592	56.1	0.109	−0.274
9	*L. tchangi*	Fang, 1936	PZ213610	16,590	56.1	0.107	−0.272
10	*Parabotia banarescui*	(Nalbant, 1965)	NC026127	16,590	56.4	0.100	−0.266
11	*Parabotia bimaculata*	Chen, 1980	MH822303	16,588	56.1	0.099	−0.267
12	*Parabotia curtus*	(Temminck & Schlegel, 1846)	LC706574	16,589	57.2	0.100	−0.272
13	*Parabotia fasciatus*	Dabry de Thiersant, 1872	NC026128	16,590	57.1	0.095	−0.267
14	*Parabotia kiangsiensis*	Liu & Guo, 1986	NC053270	16,592	55.8	0.099	−0.263
15	*Parabotia lijiangensis*	Chen, 1980	NC062659	16,595	56.1	0.098	−0.266
16	*Parabotia mantschuricus* (*Leptobotia mantschurica*)	(Berg, 1907)	NC008677	16,588	57.1	0.094	−0.266
17	*Ambastaia sidthimunki*	(Klausewitz, 1959)	NC027175	16,574	57.5	0.107	−0.268
18	*Botia dario*	(Hamilton, 1822)	MN867911	16,595	59.4	0.081	−0.237
19	*Botia lohachata*	Chaudhuri, 1912	NC027495	16,594	57.9	0.106	−0.259
20	*Botia udomritthiruji*	Ng, 2007	NC031601	16,591	57.8	0.100	−0.254
21	*Chromobotia macracanthus*	(Bleeker, 1852)	NC008671	16,937	59.1	0.103	−0.261
22	*Sinibotia pulchra*	(Wu, 1939)	NC033950	16,568	57.4	0.104	−0.262
23	*Sinibotia reevesae*	(Chang, 1944)	NC030322	16,572	57.1	−0.107	0.268
24	*Sinibotia robusta*	(Wu, 1939)	NC027853	16,575	57.5	0.109	−0.267
25	*Sinibotia superciliaris*	Günther, 1892	NC020327	16,572	57.1	0.106	−0.267
26	*Syncrossus beauforti*	(Smith, 1931)	NC031546	16,567	57.5	0.113	−0.259
27	*Syncrossus berdmorei*	Blyth, 1860	NC062730	16,553	57.6	0.108	−0.251
28	*Syncrossus hymenophysa*	(Bleeker, 1852)	NC033951	16,513	57.3	0.118	−0.274
29	*Yasuhikotakia eos*	(Taki, 1972)	NC051522	16,738	58.4	0.110	−0.265
30	*Yasuhikotakia modesta*	(Bleeker, 1864)	NC035145	16,588	58.2	0.110	−0.263
31	*Yasuhikotakia morleti*	(Tirant, 1885)	NC031600	16,567	56.7	0.125	−0.269
32	*Yasuhikotakia splendida*	(Roberts, 1995)	NC057305	16,695	56.7	0.129	−0.275
33	*Cobitis biwae*	Jordan & Snyder, 1901	NC027663	16,642	58.5	0.019	−0.211
34	*Koreocobitis naktongensis*	Kim, Park & Nalbant, 2000	NC015798	16,567	56.5	0.047	−0.236
35	*Misgurnus anguillicaudatus*	(Cantor, 1842)	NC011209	16,565	56.7	0.030	−0.220

**Table 2:** Composition and structure of *L. tchangi* mitochondrial genome.

NO.	Genes	Position (bp)	Size (bp)	Intergenic Gap (bp)	Coding Strand	Codon	
						Start	End
1	tRNA-Phe	1–69	69	0	H		
2	12S rRNA	70–1023	954	0	H		
3	tRNA-Val	1024–1095	72	0	H		
4	16S rRNA	1096–2776	1681	0	H		
5	tRNA-Leu	2777–2851	75	0	H		
6	*nad1*	2852–3826	975	8	H	ATG	TAA
7	tRNA-Ile	3835–3906	72	−2	H		
8	tRNA-Gln	3975–3905	71	1	L		
9	tRNA-Met	3977–4045	69	0	H		
10	*nad2*	4046–5091	1046	0	H	ATG	TA-
11	tRNA-Trp	5092–5160	69	2	H		
12	tRNA-Ala	5231–5163	69	1	L		
13	tRNA-Asn	5305–5233	73	30	L		
14	tRNA-Cys	5401–5336	66	1	L		
15	tRNA-Tyr	5473–5403	71	1	L		
16	*cox1*	5475–7025	1551	1	H	GTG	TAA
17	tRNA-Ser	7097–7027	71	2	L		
18	tRNA-Asp	7100–7172	73	13	H		
19	*cox2*	7186–7876	691	0	H	ATG	T--
20	tRNA-Lys	7877–7952	76	1	H		
21	*atp8*	7954–8121	168	−10	H	ATG	TAA
22	*atp6*	8112–8795	684	−1	H	ATG	TAA
23	*cox3*	8795–9579	785	0	H	ATG	TA-
24	tRNA-Gly	9580–9651	72	0	H		
25	*nad3*	9652–10,000	349	0	H	ATG	T--
26	tRNA-Arg	10,001–10,070	70	0	H		
27	*nad4L*	10,071–10,367	297	−7	H	ATG	TAA
28	*nad4*	10,361–11,742	1382	0	H	ATG	TA-
29	tRNA-His	11,743–11,812	70	0	H		
30	tRNA-Ser	11,813–11,879	67	1	H		
31	tRNA-Leu	11,881–11,953	73	0	H		
32	*nad5*	11,954–13,792	1839	−4	H	ATG	TAA
33	*nad6*	14,310–13,789	522	0	L	ATG	TAA
34	tRNA-Glu	14,379–14,311	69	4	L		
35	*cytb*	14,384–15,524	1141	0	H	ATG	T--
36	tRNA-Thr	15,525–15,596	72	−2	H		
37	tRNA-Pro	15,664–15,595	70	0	L		
38	D-loop	15,665–16,590	926	0	H		

Note: H, heavy strand; L, light strand.

**Table 3:** Base composition and size of different regions and genes in the *L. tchangi* mitogenome.

Regions	Strand	Size (bp)	T(U)	C	A	G	AT (%)	AT Skew	GC Skew
PCGs	all	11,421	27	28.8	28.9	15.4	55.9	0.033	−0.303
	H	10,899	26.3	29.6	29.6	14.6	55.9	0.059	−0.340
1st codon position	all	3807	21.1	26.2	26.7	26.0	47.8	0.118	−0.005
	H	3633	20.5	26.9	27.4	25.1	47.9	0.143	−0.035
	L	174	32.2	11.5	12.6	43.7	44.8	−0.436	0.583
2nd codon position	all	3807	40.1	27.8	18.5	13.6	58.6	−0.370	−0.341
	H	3633	39.9	28.1	18.7	13.2	58.6	−0.362	−0.360
	L	174	44.3	20.7	13.2	21.8	57.5	−0.540	0.027
3rd codon position	all	3807	19.8	32.3	41.4	6.5	61.2	0.353	−0.664
	H	3633	18.4	33.6	42.6	5.3	61.0	0.396	−0.726
	L	174	48.9	4.00	16.1	31.0	65.0	−0.504	0.770
*atp6*	H	684	28.7	29.2	28.9	13.2	57.6	0.005	−0.379
*atp8*	H	168	26.8	28.6	32.7	11.9	59.5	0.100	−0.412
*cox1*	H	1551	28.6	26.8	26.4	18.2	55.0	−0.041	−0.192
*cox2*	H	691	27.4	27.4	29.7	15.6	57.1	0.041	−0.273
*cox3*	H	785	26.1	29.8	27.0	17.1	53.1	0.017	−0.272
*cytb*	H	1141	27.0	29.9	28.8	14.3	55.8	0.033	−0.353
*nad1*	H	975	26.4	29.2	30.3	14.2	56.7	0.069	−0.348
*nad2*	H	1046	22.1	32.7	32.6	12.6	54.7	0.192	−0.443
*nad3*	H	349	29.2	29.8	27.2	13.8	56.4	−0.036	−0.368
*nad4*	H	1382	25.9	29.2	31.3	13.5	57.2	0.095	−0.367
*nad4L*	H	297	27.6	29.6	25.3	17.5	52.9	−0.045	−0.257
*nad5*	H	1839	24.7	31.0	31.5	12.7	56.2	0.120	−0.419
*nad6*	L	522	41.8	12.1	14.0	32.2	55.8	−0.498	0.455
16S rRNA	H	1681	20.1	24.0	36.2	19.8	56.3	0.285	−0.097
12S rRNA	H	954	19.0	27.0	30.7	23.3	49.7	0.236	−0.075
tRNAs	H/L	1559	26.3	22.1	28.1	23.5	54.4	0.033	0.031
D-loop	H	926	32.2	18.8	36.0	13.1	68.2	0.056	−0.182
Full genome	H	16,590	25.0	27.9	31.1	16.0	56.1	0.107	−0.272

Note: PCGs, protein-coding genes; H, heavy strand; L, light strand.

**Table 4:** Codon usage and synonymous codon bias in the *L. tchangi* mitogenome.

Codon	Count	RSCU	Codon	Count	RSCU	Codon	Count	RSCU	Codon	Count	RSCU
UUU(F)	86	0.76	UCU(S)	32	0.82	UAU(Y)	51	0.89	UGU(C)	8	0.59
UUC(F)	140	1.24	UCC(S)	67	1.71	UAC(Y)	63	1.11	UGC(C)	19	1.41
UUA(L)	104	1.00	UCA(S)	82	2.09	UAA(*)	7	4.00	UGA(W)	108	1.79
UUG(L)	16	0.15	UCG(S)	6	0.15	UAG(*)	0	0	UGG(W)	13	0.21
CUU(L)	95	0.92	CCU(P)	26	0.48	CAU(H)	25	0.46	CGU(R)	6	0.32
CUC(L)	116	1.12	CCC(P)	82	1.50	CAC(H)	83	1.54	CGC(R)	11	0.58
CUA(L)	249	2.40	CCA(P)	105	1.93	CAA(Q)	84	1.77	CGA(R)	52	2.74
CUG(L)	42	0.41	CCG(P)	5	0.09	CAG(Q)	11	0.23	CGG(R)	7	0.37
AUU(I)	176	1.19	ACU(T)	54	0.70	AAU(N)	39	0.64	AGU(S)	6	0.15
AUC(I)	120	0.81	ACC(T)	104	1.34	AAC(N)	82	1.36	AGC(S)	42	1.07
AUA(M)	126	1.52	ACA(T)	145	1.87	AAA(K)	73	1.92	AGA(*)	0	0
AUG(M)	40	0.48	ACG(T)	7	0.09	AAG(K)	3	0.08	AGG(*)	0	0
GUU(V)	53	0.97	GCU(A)	51	0.60	GAU(D)	19	0.49	GGU(G)	27	0.44
GUC(V)	43	0.79	GCC(A)	146	1.71	GAC(D)	58	1.51	GGC(G)	53	0.86
GUA(V)	95	1.74	GCA(A)	134	1.57	GAA(E)	86	1.64	GGA(G)	126	2.04
GUG(V)	27	0.50	GCG(A)	11	0.13	GAG(E)	19	0.36	GGG(G)	41	0.66

Average codons = 3807; * stop codon.

## Data Availability

The mitogenome sequence data that support the findings of this study are openly available in the NCBI GenBank at https://www.ncbi.nlm.nih.gov/ (accessed on 1 August 2026) under the accession number PZ213610. The associated BioProject, SRA, and BioSample numbers are PRJNA1444536, SRR37831197, and SAMN56759510, respectively.

## Supplementary Materials

The following supporting information can be downloaded at: https://www.mdpi.com/article/10.3390/genes17091087/s1, Table S1: Sequences of primers used in amplification of mitochondrial cytb and D-loop of *Leptobotia tchangi*; Table S2: The obtained next-generation sequencing data of *Leptobotia tchangi* in the study; Table S3: The Scaffold1 information for assembling *Leptobotia tchangi* mitogenome in the study; Table S4: The best partitioning schemes and substitution models were selected using ModelFinder v2.2.0 under the Bayesian Information Criterion (BIC) for the maximum-likelihood (ML) analysis and using PartitionFinder v2 under the Akaike Information Criterion corrected (AICc) for the Bayesian inference (BI); Table S5: The K2P genetic distance (%) between paired species within each genus of the family Botiidae; Figure S1: The structure and sequence of the control region of *Leptobotia tchangi*; Figure S2: Stem-loop structure of L-strand replication initiation region of *Leptobotia tchangi*.
