# Supplementary material for: Complete Mitochondrial Genome of the Endemic Loach Leptobotia tchangi and Its Phylogenetic Placement Within Botiidae
Source: Genes (Basel). 2026 Sep 9;17(9):1087. doi: 10.3390/genes17091087 (PMC13606129; doi:10.3390/genes17091087)
Supplement: Supplementary file 1 [file genes-17-01087-s001.zip › genes-4508673-supplementary.pdf]

Supplementary materials

1. Supplementary Tables:

Table S1. Sequences of primers used in amplification of mitochondrial *cytb* and D-loop of *Leptobotia tchangi*

Sequence	Primer name	Primer sequences (5′—3′)	*T*a/°C
CYTB	L14724	GACTTGAAAAACCACCGTTG	50
	H15915	CTCCGATCTCCGGATTACAAGAC	
D-loop	DL1	ACCCCTGGCTCCCAAAGC	55
	DH2	ATCTTAGCATCTTCAGTG	

Table S2. The obtained next-generation sequencing data of *Leptobotia tchangi* in the study

Raw data	Sample
Total Reads Count	77210458
Total Bases Count(bp)	11581568700
Average Read Length(bp)	150
Q20 Bases Count(bp)	11430945298
Q20 Bases Ratio(%)	98.70%
Q30 Bases Count(bp)	11322504440
Q30 Bases Ratio(%)	97.76%
GC content(%)	41.18%
Clean data	Sample
Total Reads Count(#)	76908928
Total Bases Count(bp)	11390793874
Average Read Length(bp)	148
Q20 Bases Count(bp)	11257928752
Q20 Bases Ratio(%)	98.83%
Q30 Bases Count(bp)	11156281433
Q30 Bases Ratio(%)	97.94%
GC content(%)	41.06%
Clean Reads(%)	99.61%
Low Quality(%)	0.32%
Too May N(%)	0.05%
Too short(%)	0.02%

Table S3. The Scaffold1 information for assembling *Leptobotia tchangi* mitogenome in the study

Parameter	Scaffold1
Length	16590
Covered_Reads_number	188090
Covered_Base_number	20841299
mean_depth	1256
Covered_percent	100
1X	16590
10X	16590
50X	16590

100X	16590
200X	16585

Table S4. The best partitioning schemes and substitution models were selected using ModelFinder v2.2.0 under the Bayesian Information Criterion (BIC) for the maximum-likelihood (ML) analysis and using PartitionFinder v2 under the Akaike Information Criterion corrected (AICc) for the Bayesian inference (BI)

Subset partitions	Best model
**ML**	
**P1:** *atp6*_codon1_*atp8*_codon1_*nad1*_codon1_*nad2*_codon1_*nad3*_codon1_*nad4L*_codon1_*nad4*_codon1_*nad5*_codon1	GTR+F+I+G4
**P2:** *atp6*_codon2_*atp8*_codon2_*cox1*_codon2_*cox2*_codon2_*cox3*_codon2_*cytb*_codon2_*nad1*_codon2_*nad2*_codon2_*nad3*_codon2_*nad4L*_codon2_*nad4*_codon2_*nad5*_codon2_*nad6*_codon2	TPM3u+F+R3
**P3:** *atp6*_codon3_*atp8*_codon3_*cox1*_codon3_*cox2*_codon3_*cox3*_codon3_*nad4L*_codon3	TN+F+I+G4
**P4:** cox1_codon1_cox3_codon1	TIM2+I+G4
**P5:** cox2_codon1_cytb_codon1	TIM2+I+G4
**P6:** *cytb*_codon3_*nad1*_codon3_*nad2*_codon3_*nad3*_codon3_*nad4*_codon3_*nad5*_codon3	TIM2+F+I+G4
**P7:** *nad6*_codon1	TN+F+I
**P8:** *nad6*_codon3	TIM3+F+G4
**BI**	
**P1:** *nad4L*_codon1, *nad4*_codon1, *nad1*_codon1, *atp6*_codon1	GTR+I+G
**P2:** *atp6*_codon2, *nad4L*_codon2	GTR+I+G
**P3:** *atp6*_codon3	GTR+I+G
**P4:** *atp8*_codon1	K80+G
**P5:** *atp8*_codon2	HKY+I
**P6:** *cox1*_codon3, *atp8*_codon3	GTR+I+G
**P7:** *cox1*_codon1	GTR+I+G
**P8:** *cytb*_codon2, *cox1*_codon2, *cox3*_codon2, *cox2*_codon2	GTR+I+G
**P9:** *cox2*_codon1, *cytb*_codon1	GTR+I+G
**P10:** *cox2*_codon3	GTR+I+G
**P11:** *cox3*_codon1	SYM+I+G
**P12:** *nad4L*_codon3, *cox3*_codon3	GTR+I+G
**P13:** *nad5*_codon3, *cytb*_codon3	GTR+I+G
**P14:** *nad4*_codon2, *nad1*_codon2, *nad3*_codon2	HKY+I+G
**P15:** *nad3*_codon3, *nad1*_codon3	GTR+I+G
**P16:** *nad3*_codon1, *nad2*_codon1	GTR+I+G
**P17:** *nad5*_codon2, *nad2*_codon2	GTR+I+G
**P18:** *nad2*_codon3	GTR+I+G
**P19:** *nad4*_codon3	GTR+I+G
**P20:** *nad5*_codon1	GTR+I+G
**P21:** *nad6*_codon1	GTR+I
**P22:** *nad6*_codon2	GTR+I+G
**P23:** *nad6*_codon3	GTR+G

Table S5. The K2P genetic distance (%) between paired species within each genus of the family Botiidae

Genus	NO.	Species sequences	Species sequences							
Ambastaia	1	*A. sidthimunki* NC027175								
			2	3	4					
Botia 10.75*	2	*B. dario* MN867911								
	3	*B. lohachata* NC027495	14.87							
	4	*B. udomritthiruji* NC031601	14.17	6.62						
Chromobotia	5	*C. macracanthus* NC 008671								
			6	7	8	8	10	11	12	13
Leptobotia 7.10*	6	*L. bellacauda* PQ822055								
	7	*L. elongata* NC018764	7.57							
	8	*L. microphthalma* NC024049	7.76	1.23						
	9	*L. pellegrini* NC031602	9.21	8.39	8.48					
	10	*L. punctata* MH644033	8.45	7.28	7.51	8.55				
	11	*L. rotundilobus* PX857782	8.52	7.52	7.71	8.69	6.84			
	12	*L. rubrilabris* NC022851	8.27	3.65	2.56	8.15	7.63	7.74		
	13	*L. taeniops* NC026130	8.94	8.07	8.15	7.39	8.15	8.58	7.17	
	14	*L. tchangi* PZ213610	9.29	8.50	8.42	7.97	8.49	8.67	7.80	6.21
			15	16	17	18	19	20	21	
Parabotia 8.11*	15	*P. banarescui* NC026127								
	16	*P. bimaculata* MH822303	9.57							
	17	*P. curtus* LC706574	10.40	10.75						
	18	*P. fasciatus* NC026128	7.92	7.14	9.78					
	19	*P. kiangsiensis* NC053270	8.18	9.20	10.08	8.13				
	20	*P. lijiangensis* NC062659	9.14	10.24	10.89	9.02	8.83			
	21	*P. mantschuricus* NC008677	8.18	6.93	9.75	2.37	8.22	9.16		
			22	23	24	25				
Sinibotia 9.04*	22	*S. pulchra* NC033950								
	23	*S. reevesae* NC030322	10.37							
	24	*S. robusta* NC027853	11.92	12.13						
	25	*S. superciliaris* NC020327	10.62	2.01	12.30					
			26	27	28					
Syncrossus 11.96*	26	*S. beauforti* NC031546								
	27	*S. berdmorei* NC062730	9.63							
	28	*S. hymenophysa* NC033951	15.04	**15.27**						
			29	30	31	32				
Yasuhikotakia 8.53*	29	*Y. eos* NC051522								
	30	*Y. modesta* NC035145	**0.16**							
	31	*Y. morleti* NC031600	12.08	12.03						
	32	*Y. splendida* NC057305	11.47	11.40	9.09					

* represent the average values of the genus.

2. Supplementary Figures:

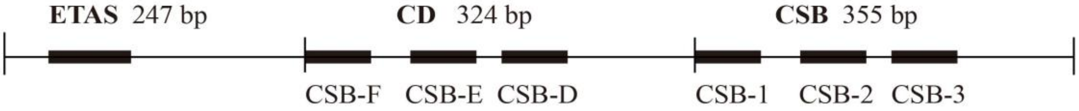

ATCTCAGCATTATGGTTTAGTACATAATATGCATAATATTACATTAATGTGCTAGTACATACTATGTATTATCACC
ETAS
AATAATTTATTTAGACCATAAAGCAAGTACTAACTTATAAGCTATACATAAGACATACATTTAAACTCAACATG
AACTCATTTTAAAATAAAGAAATAGGTCATCTACCATCACATTGTCCTCACAATTTACTTTGAAATAACCAAC
TAACATCTATGTTAGTGAATATTAATGTAGTAAGAAACCACCAACCAGTTTATATAAAGGCAAATTATTAATG
CSB-F
ATAGAATCAGGGACAATAATTGTGGGGGTCGCACAAAATGAACTATTACTGGCATCTGGTTCCTATTTCAGG
CSB-E **CSB-D**
TACATAATTGTAGAATTCCACCCTCGGATAATTATATCTGGCATTTGATTAATGGTGTAGTACATATGTCTCGTT
ACCCACCAAGCCGGGCGTTCTTTTATATGCATAACGTATCTCTTTTTGGTTTCCTTTCAATTGGCATCTCACA
GTGCAAATTCAAATAACTATCAAGGTGGATCATTTATACCTGCTTTAATTAATATAGGTTAATGATTGAAAGA
CSB-1
CATAACATAAGAGTTACATTAGTTTATCTCAAGTGCATAAGGTATATTTACTCAACACATCTTTATACTATATGC
CCCCTTTTGGTTTTCACGCGACAAACCCCCTTACCCCCTACGCTCAGAAAATCCTGTTATCCTTGTCAAACC
CSB-2
CCGAAACCAAGGAAGGCTCAACTAAGCGTACAAAGTTAACAAGTTGTAGTATGGGTTAACTTATGCCACCA
CSB-3
CATATTATATATAACATATATAAATATAACACCATAAATTATTCATCACCAAAAGCCTAAAAATTAGGATTAAAA
TTTTACGAAAAAGTCTCAATACTAAAATTTCTAAGTTCTAAATT

Figure S1. The structure and sequence of the control region of *Leptobotia tchangi*

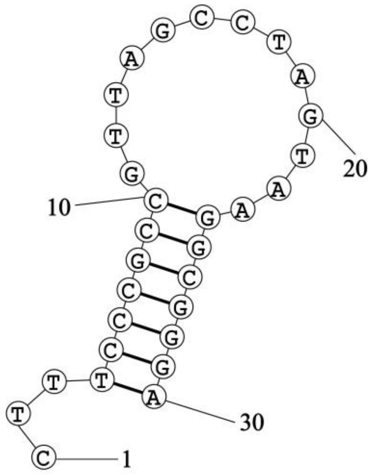

Figure S2. Stem-loop structure of L-strand replication initiation region of *Leptobotia tchangi*
